# Bacterial Zinc Metalloenzyme Inhibitors: Recent Advances and Future Perspectives

**DOI:** 10.3390/molecules28114378

**Published:** 2023-05-27

**Authors:** Riccardo Di Leo, Doretta Cuffaro, Armando Rossello, Elisa Nuti

**Affiliations:** Department of Pharmacy, University of Pisa, Via Bonanno 6, 56126 Pisa, Italy; riccardo.dileo@phd.unipi.it (R.D.L.); doretta.cuffaro@unipi.it (D.C.); armando.rossello@unipi.it (A.R.)

**Keywords:** zinc metalloenzymes, LpxC, pseudolysin, thermolysin, antivirulence agents, Gram-negative bacteria

## Abstract

Human deaths caused by Gram-negative bacteria keep rising due to the multidrug resistance (MDR) phenomenon. Therefore, it is a priority to develop novel antibiotics with different mechanisms of action. Several bacterial zinc metalloenzymes are becoming attractive targets since they do not show any similarities with the human endogenous zinc-metalloproteinases. In the last decades, there has been an increasing interest from both industry and academia in developing new inhibitors against those enzymes involved in lipid A biosynthesis, and bacteria nutrition and sporulation, e.g., UDP-[3-O-(R)-3-hydroxymyristoyl]-*N*-acetylglucosamine deacetylase (LpxC), thermolysin (TLN), and pseudolysin (PLN). Nevertheless, targeting these bacterial enzymes is harder than expected and the lack of good clinical candidates suggests that more effort is needed. This review gives an overview of bacterial zinc metalloenzyme inhibitors that have been synthesized so far, highlighting the structural features essential for inhibitory activity and the structure–activity relationships. Our discussion may stimulate and help further studies on bacterial zinc metalloenzyme inhibitors as possible novel antibacterial drugs.

## 1. Introduction

### 1.1. Bacteremia

The World Health Organization (WHO) has warned about the possibility of a new era where infections and minor wounds will be a primary cause of mortality. This emergency is correlated to the nosocomial infections (NIs) caused by the “ESKAPE” bacteria such as *Enterococcus faecium*, *Staphylococcus aureus*, *Klebsiella pneumonie*, *Acinetobacter baumannii*, *Pseudomonas aeruginosa* and *Enterobacter species* [1]. The nosocomial infections, according to the definition of WHO, indicate diseases that appear after 48 h from admission to an acute-care hospital or 30 days after receiving medical assistance, or up to 90 days after surgery [2]. Generally, the hospitalized infections are divided into four main disease-correlated sections depending on etiology: catheter-associated urinary tract infections (CAUTIs), hospital-acquired pneumonias (HAPs), bloodstream infections (BSIs), and surgical site infections (SSIs). In all these cases, pathogens may infect different districts of the human body, becoming the main cause of death of several patients [3].

The microbes constitute a real threat for both healthy individuals and patients affected by different pathologies. In order to express their pathogenic activity, bacteria firstly need to invade and colonize the potential infection sites and then to survive during blood migration. The presence of bacteria in the bloodstream is denominated bacteremia, and this phenomenon can be classified by its origin in primary and secondary bacteremia [4]. The primary bacteremia is caused by unknown sources, whereas the secondary bacteremia is a consequence of prior pneumonia or urinary infections, or of contamination due to the use of infected medical devices [5,6].

Patients affected by bacteremia often show mild (e.g., fever) or no symptoms. Nevertheless, severe infections can lead to a sepsis condition, which requires an immediate treatment in order to avoid severe consequences including death. Bacteremia is mainly caused by bacterial infection, even though several fungi could also provoke this condition [4].

It is well known that bacteria are generally classified on the basis of differences in cell membrane structure into two main categories: Gram-negative and Gram-positive. Lately, diseases associated with Gram-negative bacteria are increasing due to the growing resistance towards classical antibiotics [7]. Although different strategies have been adopted to contrast the emergency of multiple antibiotic-resistant Gram-positive and Gram-negative bacteria in hospital and community settings, novel approaches for rapid diagnosis and treatment are required.

### 1.2. Multidrug Resistance

In the last decades, microbial infections have rapidly increased due to the multidrug-resistance (MDR) phenomenon, which is the microorganism insensitivity or resistance to antibiotics [8]. Nowadays, several antibiotics are not able to combat bacterial infections (e.g., *Escherichia coli, Klebsiella pneumonia, Staphylococcus aureus* and *pneumonia, Pseudomonas aeruginosa)* (Table 1), leading to an increase of mortality and morbidity [9]. Moreover, antimicrobial resistance (AMR) can lead to high medical costs, since patients cannot be treated with classical drugs but only with more expensive therapies.

### 1.3. MDR Classification

**Figure 1:**
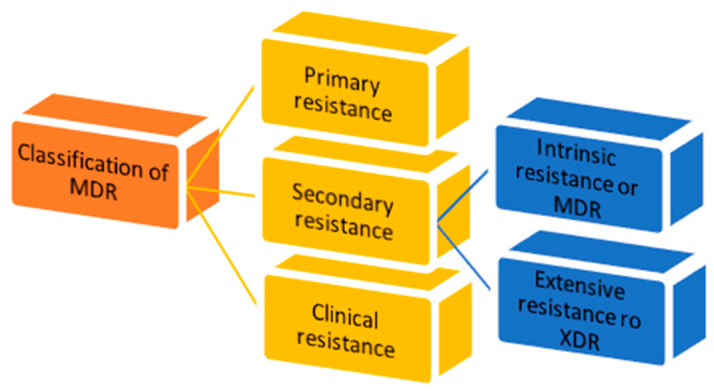
Classification of multidrug resistance.

There are three types of MDR: primary resistance, secondary resistance and clinical resistance (Figure 1) [7].

1. Primary resistance occurs when the host organism interacts for the first time with the drug of interest;
2. Secondary resistance refers to the aversion which onsets after an exposure to the drug. It is also known as “acquired resistance” and can be classified into:
 
  - intrinsic resistance: a single species of microorganisms results in insensitivity towards a first-time drug administration [11];
  - extensive resistance: microorganisms are able to resist one or more of the most potent drugs. It is also named “extended detection and response” (XDR) which arises after the exposure to first-line drugs [12];
3. Clinical resistance is described when, in order to have efficacy, the drug concentration must be significantly increased, due to dysregulation of the host immune system function. This imbalance can lead to a failure of the therapy or an outbreak of different infections [11].

### 1.4. MDR Mechanism

Microbes use different strategies in order to interfere with the activity of antimicrobial agents. These mechanisms can be intrinsic or could be acquired by gene exchange/transfer methods or genetic mutations [13].

#### 1.4.1. Modifications in the Uptake of Drugs

In order to carry out their antimicrobial activity, drugs need to reach the target site, crossing the outer membrane of Gram-negative bacteria. Bacteria are able to modulate their outer membrane functions through specific proteins named porins. Indeed, most of Gram-negative bacteria modify porin selectivity, frequency and size, interfering with the uptake of drugs [14].

#### 1.4.2. Inactivation of Drugs

Drug inactivation is one the most efficient mechanisms used by pathogens. Several bacteria use β-lactamases in order to hydrolase the β-lactam ring of classical antibiotics (penicillins and cephalosporins) into inactive compounds. Gram-negative bacteria use this mechanism to resist antibacterial agents, developing resistance. Moreover, some pathogens produce toxins as a defense mechanism, e.g., *Streptomyces* against neomycin and streptomycin [15].

#### 1.4.3. Efflux Pumps

The use of efflux pumps as a mechanism to combat the antibacterial activity of drugs is common for both Gram-negative and Gram-positive bacteria such as *Escherichia coli, Staphylococci* and *Streptococcus aureus* and *pneumoniae.* By overexpressing efflux pumps, these pathogens expel drugs outside the membrane, thus decreasing the intracellular drug concentration to a level not sufficient for activity. Moreover, some efflux pumps showed substrate-specificity against antibiotics [16].

### 1.5. Zinc Metalloenzymes

Gram-negative bacterial resistance to antibiotics is recognized as one of the main health threats in the world. Gram-negative pathogens such as *Pseudomonas aeruginosa, Escherichia coli, Acinetobacter baumannii,* and *Klebsiella pneumoniae* are associated with urinary, pneumonia and bloodstream infections [10].

The increased drug resistance in the last decades is due to the abuse of antibiotics, which enhanced the evolution of different kinds of resistance mechanisms. To date, only a few drugs have been developed against antibiotic-resistant Gram-negative pathogens [17]. Thus, there is an urgent need to develop new antibacterial agents with alternative mechanisms of action. Over the last few years, academia and industry have focused their attention on enzymes such as UDP-[3-O-(R)-3-hydroxymyristoyl]-*N-*acetylglucosamine deacetylase (LpxC), thermolysin (TLN), and pseudolysin (PLN). LpxC is a metalloenzyme involved in the biosynthesis of a key component of the Gram-negative bacterial outer membrane [1]. Otherwise, PLN and TLN are metalloproteinases secreted by Gram-negative bacteria which are involved in bacterial nutrition and sporulation [18]. Other bacterial zinc metalloenzymes taken in consideration as novel antibacterial targets are carbonic anhydrases [19,20] and histone deacetylases [21], which, however, are not considered in this review.

#### 1.5.1. Gram-Negative Outer Membrane and Its Pathogenicity

The Gram-negative bacterial cell envelope is a complex multilayered structure constituted by two main membranes, the inner and the outer, which are fundamental elements in several bacterial mechanisms. Indeed, the outer membrane comprises integral membrane proteins, phospholipids, lipopolysaccharides (LPS) and lipoproteins [22]. Interestingly, the pathogenicity of Gram-negative bacteria is correlated with the components of their outer membrane [23]. In particular, LPS are essential for Gram-negative bacteria in order to protect them from chemicals and antibiotics [23,24].

**Figure 2:**
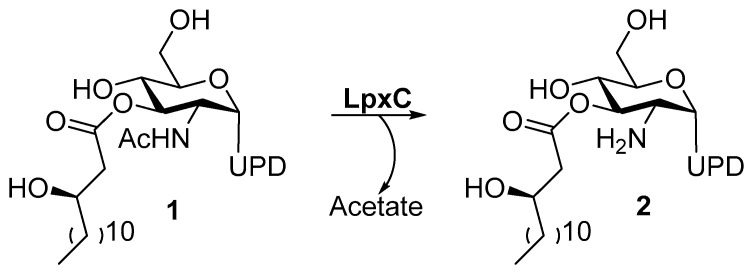
Deacetylation of UDP-3-*O*-Acyl-GlcNAc by LpxC.

LPS are constituted by three elements: lipid A, O-antigen, and core oligosaccharides. Nowadays, the research focus is on lipid A, which is the anchor for LPS in the outer membrane [25]. Lipid A is a potent immunogen that activates a severe hyperimmune response, leading to a septic shock in the host [26]. The inhibition of lipid A biosynthesis represents an attractive way to treat the Gram-negative infections and to sensitize bacteria to other drugs. The biosynthesis of lipid A involves nine unique enzymes and takes place in the cytosol of the inner membrane. Moreover, the first step of lipid A biosynthesis is thermodynamically unfavorable and it involves the LpxA enzyme [27]. Thus, the pivotal step is the second one, which requires the LpxC metalloenzyme. In particular, LpxC catalyzes the deacetylation of UDP-3-*O*-acyl-GlcNAc (**1**) through a general acid/base mechanism (Figure 2) [28].

All the enzymes involved in this pathway could be potential targets. However, LpxC is the most promising due to its role in lipid A biosynthesis, and its modulation is fundamental for *E. coli* survival. Moreover, LpxC is conserved among the Gram-negative bacteria and does not show similarities or homologies with any mammalian protein. This uniqueness made it an attractive target in order to develop selective inhibitors devoid of off-target toxicity [27].

#### 1.5.2. M4 Enzymes

Generally, proteases are able to hydrolyze proteins and peptides, and based on their activity, are classified into exo-proteases and endo-proteases. Considering their catalytic mechanism, the endo-protease family is constituted by aspartic proteases, cysteine proteases, metalloproteases, serine proteases and threonine proteases [29].

Metalloproteinases are a family of endopeptidases responsible for extracellular matrix degradation. The metalloproteinase catalytic site contains one or two metal ions (most commonly Zn^2+^). The divalent ions play a fundamental role in the catalytic mechanism by activating a nucleophilic water molecule, exploiting the protease mechanism. Furthermore, several calcium ions are located in the binding site and do not participate in the catalytic activity [30].

According to the MEROPS database (http://MEROPS.sanger.ac.uk/ accessed on 9 March 2023), metalloproteinases are classified into 76 families (M1-M76), which are divided into 16 clans (MA, MC, MD, ME, MF, MG, MH, MJ, MM, MN, MO, MP, MQ, MS, MT and unassigned) based on their similarities to the 3D structure and metal ion motifs. The clan is indicated by two letters; the first letter indicates the family which the clan is referred to ((M) Metalloproteinase). However, sometimes some families cannot yet be assigned to clans and when this happens the family is described as belonging to clan A-, C-, M-, S-, T- or U-, according to the catalytic type. Moreover, some clans are divided into sub-clans due to the age differences within the clan [31].

Most of the virulence factors involved in bacterial nutrition and sporulation (e.g., TLN and PLN) belong to the M4 family, in particular to the clan MA, sub-clan E. M4 metalloproteinases are extracellular proteases secreted by bacteria, and are fundamental in the protein degradation to provide nutrients for bacteria. Among MA metalloproteinases, Thermolysin (TNL) from *Bacillus thermoproteolyticus* has been the first M4 protease to be identified and it represents the prototype of this family [18].

The M4 enzymes are characterized by the presence of a Zn^2+^ ion in the catalytic site coordinated by two histidine residues, one glutamic acid and one water molecule constituting the HEXXH motif. These proteases are secreted as pre-propeptides constituted by a signal peptide, a propeptide sequence and a peptidase unit. Furthermore, the 3D structure is composed of two domains and the binding site is located between them [32]. The *N-*terminal domain includes α-helices and β-sheets, while the *C*-terminal is formed by α-helices connected by a turn. In this region, the glutamic acid residue coordinates the zinc ion [33].

Generally, the binding site of a metalloproteinase contains different subsites (S1, S2, S3 and S1′, S2′ and S3′). Among them, the S1′ subsite is crucial in substrate recognition because it presents a different shape in each metalloproteinase. In particular, the S1′ pocket interacts with hydrophobic or bulky side chains of amino acids constituting a hydrophobic site. The S2′ subsite is a hydrophobic pocket, whereas the S1 subsite is characterized by hydrophilic residues [34]. Generally, the hydrolysis of the substrates is based on an acid-base mechanism. In particular, a histidine residue present in the binding site reacts as a base that activates the water molecule. The formed hydroxyl group attacks the carbonyl group of the substrate, leading to the cleavage.

The M4 enzymes are structurally similar to the endogenous Metzincins, i.e., Matrix metalloproteinases (MMPs) and A disintegrin and metalloproteinases (ADAMs) [35,36]), which are classified into the M10 family. Proteases of the M10 family have a Zn^2+^ in the catalytic site coordinated by three histidine residues and one water molecule. Moreover, in the binding pocket the MMPs present the same subsites of the M4 enzymes [37]. However, the particular depth of the S1′ subsite might represent a selectivity factor between the M4 and M10 enzymes [38].

### 1.6. Targets of Interest

#### 1.6.1. UDP-[3-O-(R)-3-hydroxymyristoyl]-*N*-acetylglucosamine Deacetylase

UDP-[3-O-(R)-3-hydroxymyristoyl]-*N*-acetylglucosamine deacetylase (LpxC) is a cytosolic zinc metalloenzyme which catalyzes the second step in the biosynthesis of lipid A (Figure 2), a key component of all Gram-negative bacterial outer membranes. Thus, the inhibition of its biosynthesis can lead to an increase of cell membrane permeability and leakage of cell contents up to cell death, as previously described [28].

##### Structure of LpxC

**Figure 3:**
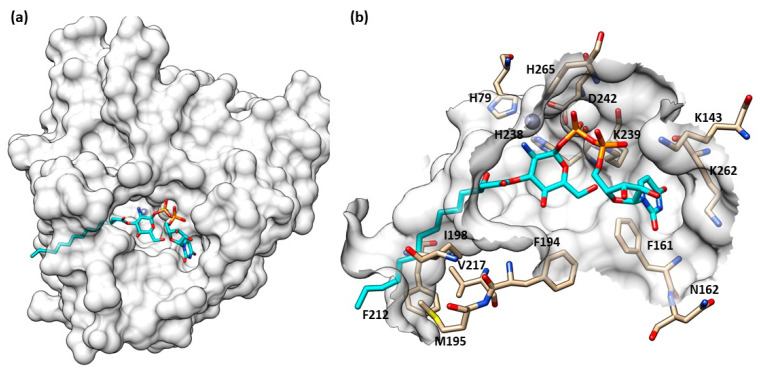
(**a**) Crystal structure of compound **2** in LpxC (PDB ID: 4MDT) of *E. coli.* (**b**) Compound **2** into LpxC (PDB ID: 4MDT) binding site of *E. coli*. Image generated with Chimera, version 1.16.

Structurally, LpxC is constituted by two topologically similar domains, presenting two α-helices packed against a five-stranded β-sheet, the active site being located at the interface between them [17]. The Zn^2+^ displayed at the bottom of the site is coordinated by two histidines (His79 and His238), one aspartate residue (Asp242) and one water molecule, preserving its classical tetrahedral coordination geometry. A crystal structure of *E. coli* LpxC in complex with the deacetylated compound **2** (Figure 2 and Figure 3) shows the different binding regions of the conical-shaped active site. The fatty acyl moiety of compound **2** interacts with the hydrophobic tunnel composed of Met195, Ile198, Phe212 and Val217. The hexose ring fragment of **2** is placed in a slightly hydrophobic pocket comprising Phe161, Phe162 and Phe194 residues. Lastly, the UDP-binding pocket is composed of a phosphate binding region surrounded by polar residues (Lys143, Lys239, Lys 262 and His265) and a nucleotide binding site, where the UDP moiety forms polar (Asp160) and π-stacking interactions (Lys262 and Phe161) [39].

#### 1.6.2. Pseudolysin

Pseudolysin (LasB elastase or elastase B) is a virulence factor produced by *P. aeruginosa* and can be considered a novel target in the fight of nosocomial infections. This protease belongs to the thermolysin-like family of metallopeptidase (M4 family) and displays a crucial role in the pathogenesis of infections caused by *P. aeruginosa* [40]. Pseudolysin hydrolyses several host proteins useful for bacterial nutrition, leading to a disorder of the immune response and encouraging inflammatory processes.

Pseudolysin is involved in different diseases, such as the destruction of laminae of the arteriae in vasculitis combined with the action of staphylolysin [41]. Moreover, it causes hemorrhage, destroying the basement membrane of vessels [42]. Pseudolysin causes septic shock in septicemia and supports the growth and invasiveness of microbes in burns [43,44]. In lung diseases, this enzyme is involved in the damage of alveolar septal cells and, by destroying the alveolar epithelial cell junctions, it supports the epithelial permeability to macromolecules [45]. Moreover, pseudolysin is involved in the cystic fibrosis caused by *P. aeruginosa* [46]. Thus, it can be considered a good target to combat several diseases.

##### Structure of Pseudolysin

Pseudolysin is synthesized as a preproelastase with 498 residues and is composed of three domains. The *N*-terminal prepeptide or signal peptide (aa 1–23) is cleaved across the cytoplasmic membrane, giving the proenzyme. The second domain, the propeptide domain (aa 24–197), shows an intramolecular chaperone (ICM) activity for the *C*-terminal domain (aa 198–498) in order to encode the mature enzyme. The mature elastase has 67% homology with thermolysin from *Bacillus thermoproteolyticus.* Furthermore, the expression of pseudolysin is regulated by four sophisticated gene regulation systems (LasI-LasR, Rh1I-Rh1R, PqsABCDE-PqsR and AmbCDE-IqsR) that depend on the bacterial cell density. These systems belong to a complex Quorum sensing (QS) regulatory network [47]. *Pseudomonas* is also usually associated with biofilm infections because the biofilm formation is regulated by the QS [48].

**Figure 4:**
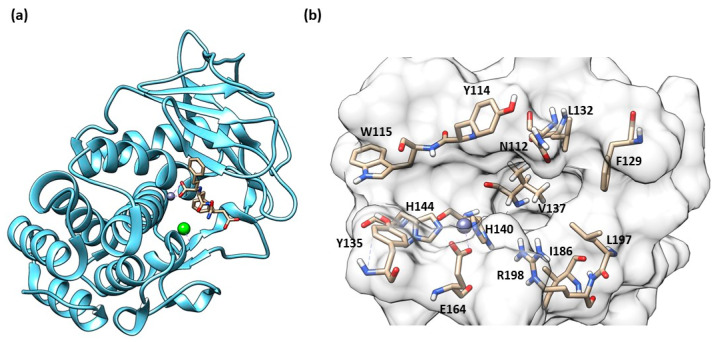
(**a**) Secondary structure of Pseudolysin (PDB ID: 1U4G). (**b**) Amino-acids constituting the subsites of pseudolysin (PDB ID: 1U4G). Image generated with Chimera, version 1.16.

The secondary structure of the protein is constituted by seven α-helices and two antiparallel β-sheets. The binding site is allocated among two helices and their connective loop (Figure 4a). Pseudolysin contains a zinc and a calcium ion [49]. The Ca^2+^ is useful for protein stability, whereas the Zn^2+^ close to the cleavage site plays a catalytic role [50]. Two disulfide bridges (Cys30-Cys58 and Cys270-Cys297) are useful for the protein stabilization. The tetrahedral coordination of Zn^2+^ is formed by two histidines (His140 and His144), one glutamate (Glu164) and a catalytic water molecule. Furthermore, the protease presents three significant pockets, as already mentioned in paragraph 1.5.2: S1′, S2′ and S1 (Figure 4b). The S1′ pocket recognizes aromatic and hydrophobic amino acids including Leu132 and Val137. The S2′ subsite contains Asn112, Phe129, Ile186 and Leu197 and interacts with hydrophobic residues too. In sharp contrast, the largest pocket, S1, composed of hydrophilic residues (Tyr114, Trp115, Tyr 155), interacts with hydrophilic moieties. The S1′ subsite is the most important for selectivity and activity; it is smaller than the S1′ subsites of human MMPs due to the Arg198 placed between the S1′ and S2′ pockets, which regulates the size and the entrance of the pocket [51].

#### 1.6.3. Thermolysin

Thermolysin (TLN) from *Bacillus thermoproteolyticus* is the enzyme prototype for the M4 family of metalloproteinases and represents another innovative target against bacterial infections. This enzyme is used in the biotechnology industry to cleave amino-peptide bonds and it is also utilized for the production of aspartame. It has peculiar characteristics, being a thermozyme: it is stable in extreme temperatures, pH values and is also stable in organic solvents [32]. For these reasons, it is used for different industrial applications. Thermolysin is involved in the development of peptic ulcer, gastritis, gastric carcinoma and cholera. The thermolysin-like proteinase from Legionella is suggested to be responsible for Legionnaires’ disease and pneumonia [18].

##### Structure of Thermolysin

**Figure 5:**
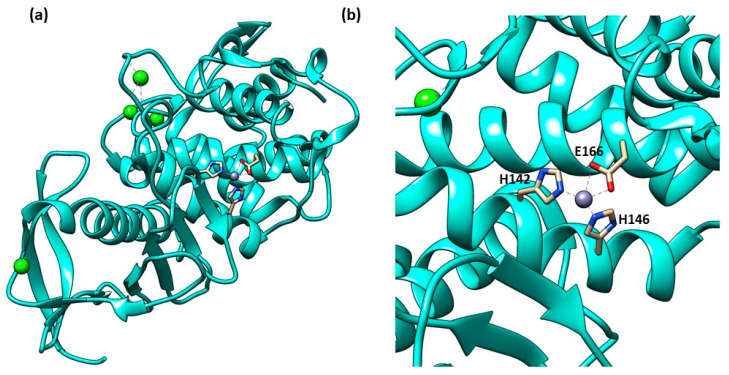
(**a**) Secondary structure of TLN (PDB ID: 1PE5); (**b**) Tetrahedral coordination of catalytic zinc ion in TLN (PDB ID: 1PE5). Image generated with Chimera, version 1.16.

Thermolysin’s three-dimensional structure presents one catalytic zinc ion in the binding site and four calcium ions (Figure 5a). The catalytic zinc, like all the M4 enzymes, has a tetrahedral coordination composed of two histidines, a glutamatic acid and one water molecule (Figure 5b). Pseudolysin’s structure shares 67% of homology with thermolysin. However, the binding site cleft of pseudolysin is slightly wider than the thermolysin binding site cleft. Moreover, the physics profile to interact with hydrophilic or hydrophobic residues of the subpockets (S1′, S2′ and S1) are the same between the two proteinases. The size of the S1′ subpocket of thermolysin, the main subsite for the inhibitor recognition, is slightly smaller than pseudolysin.

## 2. Inhibitors

The increase of nosocomial infections due to the “ESKAPE” pathogens is becoming an emergency and the antibiotic resistance phenomenon is its main cause. According to the World Health Organization (WHO), the discovery of novel enzyme inhibitors able to interfere with bacterial infections by avoiding the classical resistance mechanism could have an important role [1]. 

The high level of similarities among these metalloproteinases makes it challenging to selectively target M4 metalloproteinases. In fact, the crucial features for an effective inhibitor of metalloproteinases are the presence of a zinc-binding group (ZBG) and a hydrophobic group able to interact with the S1′ pocket. The ZBG is a moiety able to coordinate the catalytic zinc ion and the most reported ZBG is hydroxamate, even if it is responsible for important adverse effects and low selectivity [52]. More often, a good fitting with the S1′ pocket allows the use of alternative ZBGs such as carboxylate [53] or allows the development of non-zinc-binding inhibitors [54].

These elements are common to all the already reported inhibitors of metalloproteinases either of bacterial (such as M4) or human origin, including MMPs [55,56,57] and ADAMs [58,59]. Nowadays, the real challenge is to obtain selective M4 metalloproteinase inhibitors sparing the human endogenous metalloproteinase as the key factor to avoid several side effects.

This review aims to collect and classify the potential inhibitors developed from 2015 to the present against the most studied bacterial zinc metalloenzymes (LpxC, PLN and TLN), correlated to Gram-negative bacteria. In particular, the structural features of ligands essential for achieving inhibitory activity and their structure-activity relationships will be described. All reported compounds will be classified based on their chemical structure and ZBG.

### 2.1. LpxC inhibitors

#### 2.1.1. Hydroxamate-Based LpxC inhibitors

##### CHIR-090 and Its Analogues

**Figure 6:**
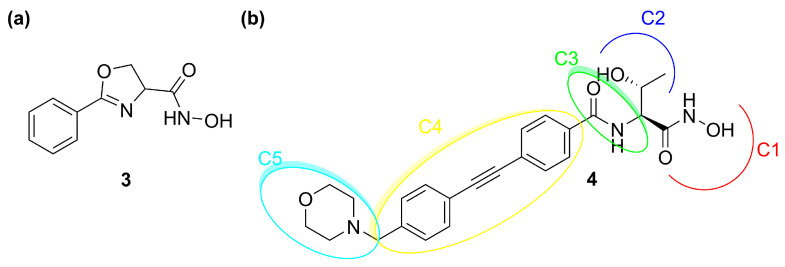
(**a**) Structure of **3** (L-573,655). (**b**) Structure of **4** (CHIR-090).

The development of LpxC inhibitors took place following the discovery of deacetylase inhibitor L-573,655 (**3**, Figure 6a) at Merck in 1996. Then, different pharmaceutical companies such as Chiron, Astra-Zeneca, and Pfizer accepted the challenge to develop inhibitors against the deacetylase enzyme [60].

So far, none of the developed compounds have reached the market and the most studied inhibitor of LpxC is CHIR-090 (**4**), which showed excellent in vitro potency against several Gram-negative pathogens. The analysis of its chemical structure allowed researchers to identify a pharmacophore with five necessary sites for the enzyme–inhibitor interaction (Figure 6b) [61].

C1 moiety is the ZBG which coordinates the Zn^2+^ in the catalytic site. C2 is the site for several substituents and C3 is a linker between the “head” of the inhibitor and the lipophilic “tail” (C4). The latter interacts with the hydrophobic tunnel of the enzyme. The last site is C5, which interacts with a solvent-exposed pocket and can influence the ability of the molecule to cross the bacterial membrane.

Moreover, the efficacy of **4** was tested alone and in co-administration with colistin against *P. aeruginosa* biofilm. Colistin (polymyxin E) is a natural antibiotic which interacts with the LPS, causing increasing penetration of substances from the outer membrane of the Gram-negative bacteria. The combination of colistin and **4** demonstrated a synergistic activity against both colistin-susceptible and colistin-non-susceptible *P. aeruginosa* biofilms. The mechanism of this synergism has not been explained yet [62].

**Figure 7:**
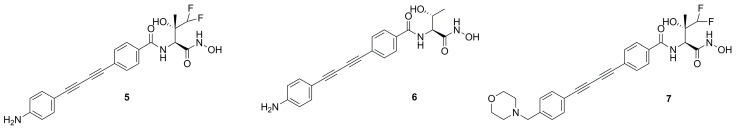
Structures of **5**, **6** and **7**.

Titecat et al. in 2016 evaluated the antibiotic activity of three deacetylase inhibitors (**5**, **6** and **7**) against Gram-negative bacilli and investigated the effect of these inhibitors in combination with conventional antibiotics (Figure 7) [63].

The best compound of this series and the first inhibitor to have activity against both *A. baumannii* and *B. cepacian* was identified as **5** [64]. Moreover, **5** was also active against MDR/XDR Enterobacteriaceae and *P. aeruginosa* strains and demonstrated a synergy with conventional antibiotics. The greater potency and wider spectrum of activity displayed by these compounds were due to the presence of the biphenyl–acetylene moiety which is supposed to interact within the hydrophobic tunnel of the enzyme. The difluoromethyl fragment might increase the ability to cross the lipid barrier and it might justify the activity of both **5** and **7** against *A. baumannii*. Furthermore, Gram-negative bacteria are known to be capable of excreting compounds containing a morpholine group through the efflux pump, which could explain the major activity of **5** with respect to morpholino-derivative **7**.

**Figure 8:**
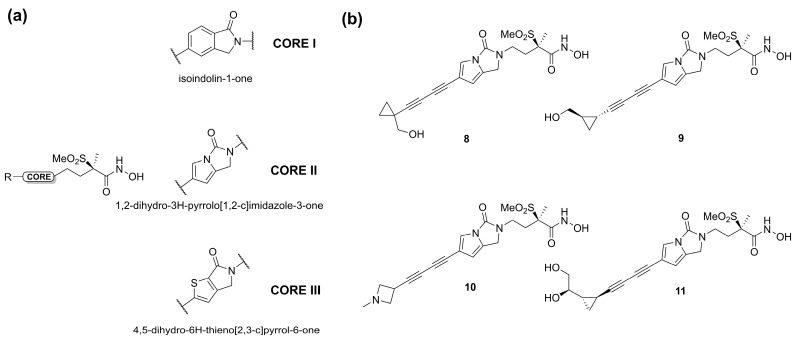
(**a**) Core structures of methylsulfone hydroxamate LpxC inhibitors. (**b**) Chemical structure of Surivet inhibitors.

In 2020, Surivet et al. [65] tried to identify novel methylsulfone hydroxamate LpxC inhibitors using three different cores (Figure 8a). The pyrrolo-imidazole series (core 2) displayed a good activity against *E. coli* in a murine infection model, especially compounds **8** and **9** (Figure 8b). Unfortunately, unattainable human doses of these compounds were necessary in order to exhibit their efficacy *in vivo*. Moreover, compounds **8** and **9** displayed solubility issues unfavorable to a future intravenous administration. Therefore, the terminus of the hydrophobic fragment of **8** was modified with polar substituents to improve its bioavailability by intravenous administration [66]. As expected, compounds **10** and **11** (Figure 8b), containing an amine and a diol group, respectively, showed high solubility and potency against Gram-negative bacteria in animal infection models (Table 2).

**Figure 9:**
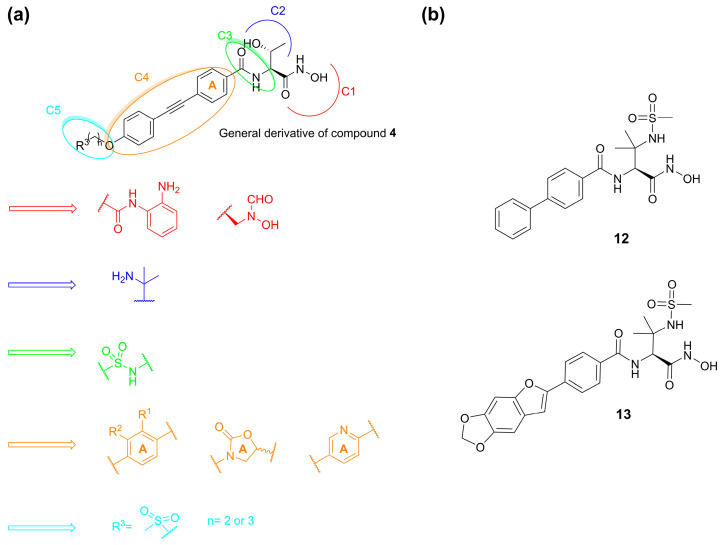
(**a**) SAR developed by Ding et al. (**b**) Structures of compounds **12** and **13**.

Ding et al. in 2018 synthesized several series of derivatives of compound **4** (Figure 9a) and tested their antibacterial activities against *Escherichia coli* (PA01, PA14) and *P. aeruginosa* (AB1157, DH5α) in vitro strains. Moreover, their metabolic stability was evaluated in liver microsomes. Due to its weak metabolism, the hydroxamic acid was replaced by acyl *o*-phenylenediamine and reversed hydroxamic acid. Unfortunately, these modifications turned out to be unsuccessful. Then, the introduction of a sulfonamide in the C3 site did not increase the antibacterial activity; instead, the metabolic stability in the human liver microsomes was improved with the replacement of the 2-hydroxy ethyl group by the 2-amino isopropyl group in the C2 site [61]. Moreover, in the C4 site the introduction of substituents on the benzene ring or its replacement with oxalidinone or pyridine ring decreased the inhibitory activity. Furthermore, the lengths of the hydrophobic scaffold and the hydrophilic terminus were investigated and the compounds bearing diphenylacetylene moiety in C4 and a methylsulfone group in C5 exhibited comparable activities to **4** against the classical Gram-negative pathogen strains (Figure 9a) [67].

**Figure 10:**
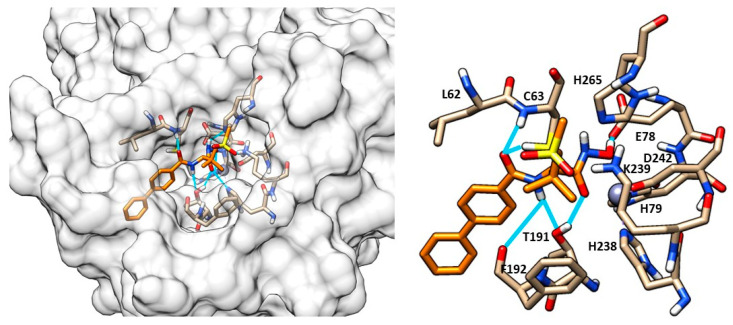
Docking of compound **12** (orange) into LpxC (PDB ID: 3P3G). Image generated with Chimera, version 1.16.

Another study by Kawai et al. identified a sulfonamide-based hydroxamic acid **12** (Figure 9b) that was more active on *E. coli* and *P. aeruginosa* than on *K. pneumonia*. The docking of **12** in *E. coli* LpxC showed the chelation of the Zn^2+^ by the hydroxamic acid. Furthermore, the oxygen atom of the amide made dual contacts with the backbone NH and the thiol on side chain of Cys63. Instead, the NH of the amide interacted with the hydroxyl group of Thr191 and the backbone carbonyl of Phe192. Finally, the biphenyl moiety was inserted in the hydrophobic tunnel (Figure 10). In order to improve the potency and the antibacterial spectrum of **12**, the biphenyl group was replaced by different longer non-alkyne hydrophobic moieties and the analogue compound, **13**, bearing a tricyclic fragment displayed the best activity against *E. coli, K. pneumoniae* and *P. aeruginosa* (Table 3) [68].

**Figure 11:**
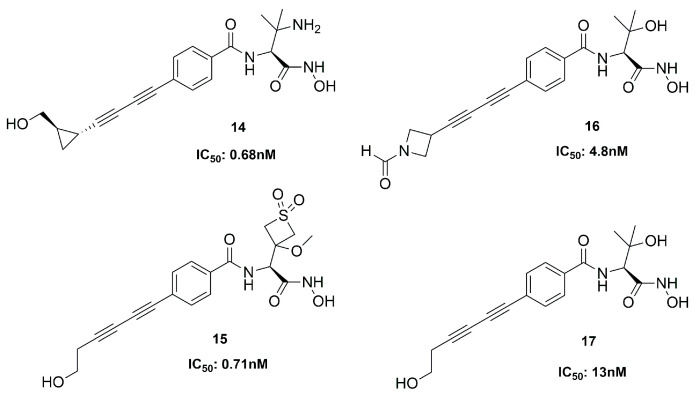
Inhibitors developed at Achaogen.

At Achaogen Inc., different LpxC inhibitors with a bis-alkyne scaffold against multidrug-resistant (MDR) *Pseudomonas aeruginosa* strains were studied. This bacterium is the most common cause of chronic infections in patients with cystic fibrosis (CF). The newly developed compounds (**14**–**17**, Figure 11) were tested in healthy volunteers to evaluate their safety, tolerability, and pharmacokinetics (PK).

Compound **14** was the first inhibitor to be evaluated in Phase I clinical trials, but its therapeutic window was insufficient. Then, three analogues were analyzed, and they showed a wider therapeutic window than **14.** All of them were potent inhibitors of the deacetylase; in particular, **15** was the best, due to its favorable activity, safety and PK profile [69]. Unfortunately, since all these compounds displayed cardiovascular toxicity in preclinical models at any efficacious dose, the study could not be further developed [70].

**Figure 12:**
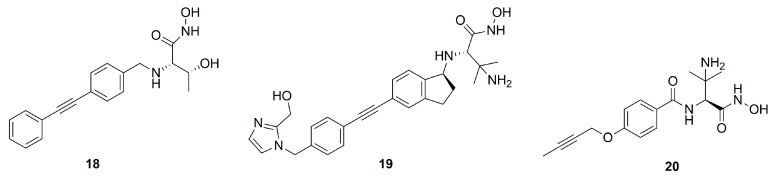
Structures of compounds **18**, **19** and **20**.

The bicyclic derivative **19** (Figure 12) was developed by Zhang et al., starting from the promising threonyl compound **18**, which showed an IC_50_ < 100 nM towards different Gram-negative pathogens. Compound **19** kept the broad-spectrum activity against Gram-negative bacteria, showing a good PK profile in three different animal species (rat, mice and monkey, IV and SC). However, despite this positive profile, **19** was not a good candidate for further clinical trials due to the observed acute tolerability problem associated with the inhibition of sodium channels [71].

LpxC inhibitors with a short hydrophobic group as a tail were synthesized by Novartis researchers. These new compounds should have a low PPB (plasma protein binding) and high solubility, with significant activity and low cytotoxicity. Moreover, the Novartis inhibitors should be selective against the *P. aeruginosa* LpxC enzyme in order to fight its infections and at the same time prevent the antibacterial cross-resistance and the unintentional disturbance of the human microbiome usually caused by broad-spectrum antibiotics. Indeed, the lack of the di-acetylene moiety together with its replacement with a short propargyl group led to derivative **20** (Figure 12) endowed with enhanced specific inhibition of *P. aeruginosa* LpxC. In particular, the specificity was achieved thanks to the “gatekeeper” helix and neighboring residues (aa 192−205) of the hydrophobic tunnel region, which adapted the channel to different inhibitor “tails”. Inhibitor **20**, the best of the series (IC_50_ = 1.5 nM), showed a good efficacy in vitro and excellent results in in vivo studies too. These results would warrant further in vivo investigations to fully understand PK/PD parameters and subsequent human dose predictions [60].

##### Oxazolidinones Derivatives

**Figure 13:**
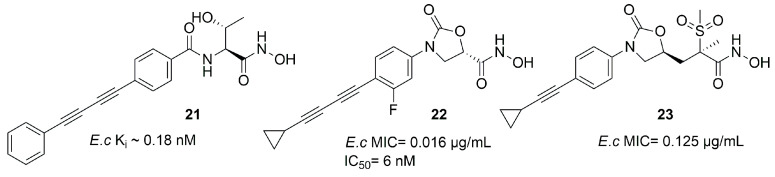
Compound **21**, **22** and **23** structures (*E.c*: *Escherichia coli*).

Kurasaki et al. in 2016, starting from **4** (Figure 6b) and **21** (Figure 13), developed a series of hydroxamate-based compounds endowed with a good activity against *E*. *coli* and *K. pneumoniae* LpxC enzymes. Based on the crystal structure of **21** in the binding site of *E. coli* LpxC, the authors showed that the carbonyl group of the benzamide moiety lay in the same plane of the threonyl fragment. Thus, they combined the threonyl group and the benzamide moiety of **4** into an oxazolidinone cycle while the *S*-configuration of the ZBG was kept. In order to increase the affinity with the hydrophobic tunnel of the enzyme, the biphenyl acetylene moiety was replaced with a diene fragment. Moreover, the effect of substituents on the central phenyl ring led to a decrease in the activity against *Pseudomonas aeruginosa* and *Acinetobacter baumannii* [72]. Based on these considerations, the best compound of the series was the inhibitor **22** (IC_50_ = 6 nM, Figure 13), which showed a nanomolar activity against *E. coli* and *K. pneumoniae* and a low efflux ratio [73].

Moreover, compounds bearing an oxazolidinone or isoxazoline fragment as a linker between the ZBG and the hydrophobic moieties were selected by computational approach. Thus, two series of compounds were synthesized and tested against Lpxc enzymes from *E. coli*, *P. aeruginosa* and *K. pneumoniae* and both of them displayed similar inhibitory activity. However, the goal was reached by methylsulfone **23**, which contained the oxazolidinone moiety (Figure 13) and proved to have a good activity against all the three bacteria. Furthermore, the activity against *K. pneumoniae* could be improved by a combination with Gram-positive antibiotics such as rifampicin and vancomycin, both in in vitro and in vivo experiments [74].

##### *C*-Furanose Derivatives as LpxC Inhibitors

**Figure 14:**
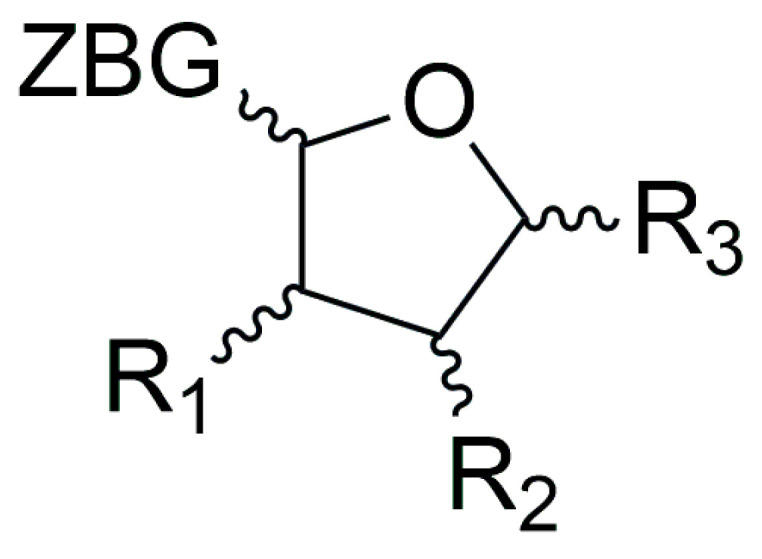
General structure of furanoside inhibitors.

To this class of LpxC inhibitors belong compounds with a furanose ring or an acyclic analogue moiety which bear different long hydrophobic fragments in 5 position (R_3_) in order to interact with the hydrophobic tunnel of the enzyme. The 1 position is occupied by the ZBG, which is usually a hydroxamic acid. The 3 and 4 positions present hydrophilic moieties (Figure 14). Moreover, the importance of each chiral carbon was investigated by different research groups.

**Figure 15:**
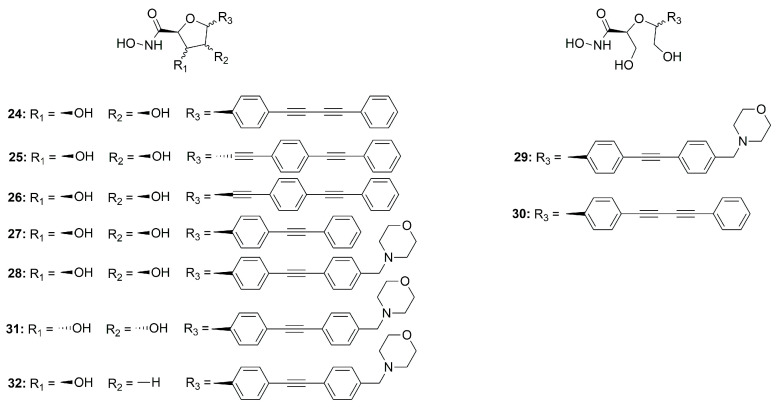
Sugar-derivative inhibitors **24**–**32**.

In 2015, Jana et al. synthesized a series of *C*-ethynyl furanoside LpxC inhibitors based on compound **4** structure. The 1-hydroxyethyl fragment of **4** was incorporated in the sugar moiety and the amide group was replaced by an ether group. Starting from compound **24** (Figure 15) (IC_50_ = 30.5 ± 4.2 µM, *K*_i_ = 4.2 ± 0.6 µM), the lipophilic side chain length and its correct orientation were analyzed focusing on the anomeric center of the sugar core. Compound **25** (Figure 15) was the only one active against *E. coli* (D22) LpxC with a *K*_i_ = 14.7 µM and IC_50_ = 107 ± 12 µM. Its β-anomer **26** (Figure 15) (IC_50_ > 200 µM) did not show any inhibitory activity (Figure 15). Thus, the α configuration proved to be necessary for efficacy [75].

**Figure 16:**
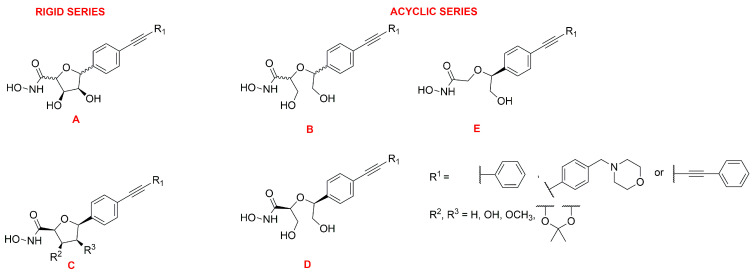
General representation of cyclic (A and C series) and acyclic (B, D and E series) sugar-based inhibitors.

Müller et al. developed a SAR study on *C-*furanoside LpxC inhibitors, synthesizing and analyzing different series of compounds among which are derivatives **24**, **27** and **28** (Figure 15 and Figure 16). It is known that compounds with a diphenylacetylene group as a lipophilic side chain have shown the worst inhibitory activity due to their shorter hydrophobic fragments that cannot occupy the entire hydrophobic tunnel of the enzyme. Morpholinomethyl-substituted diphenylacetylene derivatives displayed the highest antibacterial activities evaluated through the disc diffusion test, although their inhibitory activity was slightly weaker than that of the respective diphenyldiacetylene analogues. Furthermore, the data suggested that the 2,5-*trans*-configured *C*-glycosides (A series were more efficient than their respective *cis*-stereoisomers (C series). In particular, the best configuration was 2R, 5S. An improvement of the activity was pointed out by methylation of the hydroxyl group in 4 position and formation of the isopropylidene acetal (B series). However, the series of the acyclic diols displayed better biological activities than the respective *C*-glycosides, although they were not rigid compounds (B and C series). The open-chain (2S,5S)-configured compounds **29** and **30** (Figure 15) were the most potent LpxC inhibitors of these series of inhibitors (Table 4). Moreover, an improvement of the inhibitory activity was observed with the removal of the hydroxyl group in 3 position too (E series) [76].

The *C*-glycosides compounds were slightly weaker than the acyclic analogues because a hydroxyl group was located in a non-polar region of the binding site. Hence, the structure of compound **28** (Figure 15) was optimized by inverting the configuration in positions 3 and 4 of the rigid system. The new stereoisomer **31** (Figure 15) exhibited the best inhibitory activity against *E. coli* BL21 (DE3) and the D22 strain [39]. This improvement was due to the orientation of the hydroxyl group towards the entrance of the catalytic site of the enzyme, where it formed water-mediated hydrogen bonds with surrounding amino acids. The stereoisomer **31** showed the same activity of the respective (S, S)-acyclic compound **29** (Figure 15), suggesting that modification in positions 3 and 4 will be necessary in order to improve the activity (Table 4).

Dreger et al. have investigated the capacity of *C*-glycosides inhibitors to reach their cytoplasmatic target by crossing the bacterial cell wall. The study revealed that further optimization would be necessary to improve the permeability of the sugar-core inhibitors [39].

Later, monohydroxy-tetrahydrofuran analogues of the rigid series were analyzed by the same group. The removal of the hydroxyl group in 3 position did not increase the inhibitory activity, whereas the elimination of the hydroxyl group in position 4 led to an improvement of the efficacy. Compound **32** (Figure 15), with a (2S, 3R, 5R) configuration, exhibited the highest antibacterial activity against *E. coli* BL21 (DE3) and the D22 strain (Table 4) [77].

**Figure 17:**
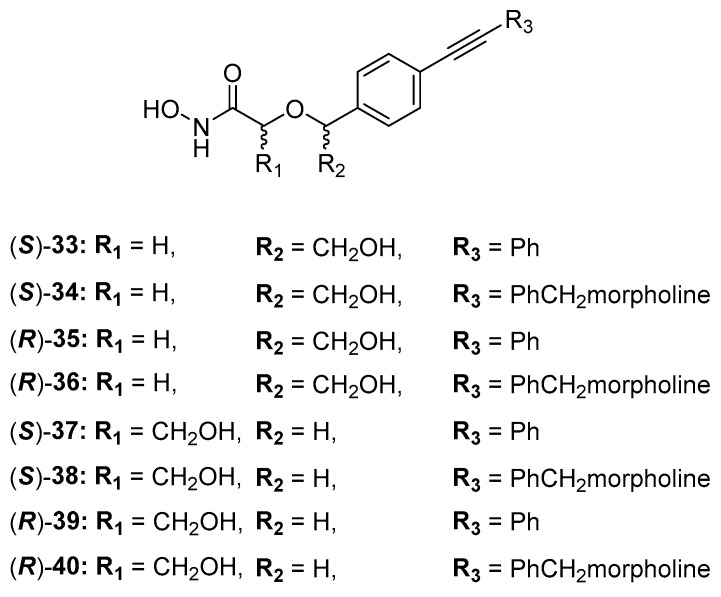
Structures of acyclic derivatives of series E.

The acyclic series E was investigated by Tangherlini et al. in 2016, starting from compound **29** (Figure 15). The researchers demonstrated that the shift of the hydroxylmethyl group from the α-hydroxamic acid position (**37**–**40**, Figure 17) to the benzylic fragment (**33**–**36**, Figure 17) slightly increased the efficacy against the *E. coli* LpxC enzyme, whereas configuration in α-position to the hydroxamic acid did not have a relevant influence. Indeed, the *K*_i_ values of **37** and **38** were similar to those of their respective enantiomers **39** and **40**. (Figure 17, Table 5) [78].

**Figure 18:**
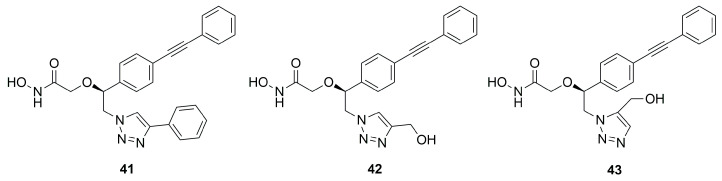
Inhibitors bearing a triazole ring as a linker.

Further optimization of compound **33** was carried out by replacing its hydroxyl group with a triazole ring in order to keep the possibility of forming hydrogen bonds in the catalytic site of the enzyme. Moreover, the triazole ring was used as a linker to connect other substituents which could interact with the unoccupied region of the UDP-binding site. Compounds **41** (IC_50_ = 10.4 ± 9.7 µM) and **42** (IC_50_ = 8.5 ± 1.3 µM) (Figure 18) bearing a phenyl ring and a hydroxymethyl group, respectively, in position 4 of the triazole, and inhibitor **43** (IC_50_ = 23.4 ± 5.8 µM) with a hydroxymethyl group in position 5 of the triazole, were the best of the series, showing the biggest halos of inhibition in the disc diffusion assays [79].

In particular, docking studies revealed that the increased activity should be attributed either to the aromatic interactions between the tetrazole ring and Phe192 or the interactions between the tetrazole ring substituent with Lys239. However, these compounds displayed a lower activity with respect to the most studied LpxC inhibitor **4**.

In a following study, the hydroxamic acid of compound **33** (Figure 17) was replaced by different ZBGs due to its unfavorable pharmacokinetic properties. However, all new derivatives displayed a loss of LpxC inhibitory and antibacterial activity [80].

#### 2.1.2. Non-Hydroxamate LpxC Inhibitors

The discovery of inhibitors without a hydroxamate group as ZBG represents an interesting approach in order to avoid side effects due to the lack of specificity previously seen in many matrix metalloproteinase (MMP) inhibitors [38]. Moreover, the hydroxamate group is typical of HDAC inhibitors [81] but also represents the main cause of their mutagenicity [82]. The strategies to avoid the use of hydroxamic acids are fundamentally three: use of alternative ZBGs, binding to exosites [52,83] and exploiting an excellent fitting with the S1′, S1 and S2 pockets of the catalytic site.

**Figure 19:**
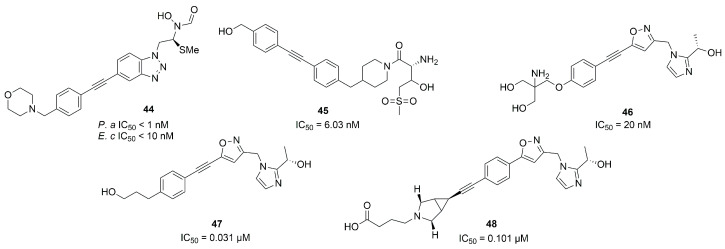
Non-Hydroxamate LpxC inhibitors (*P.a*: *Pseudomonas aeruginosa*, *E.c*: *Escherichia coli*).

Furuya et al. in 2020 synthesized a series of LpxC inhibitors bearing a *N*-hydroxyformamide (NHF) as ZBG. Based on the structure of compound **4**, they developed different derivatives with a benzotriazole as a core and several substituents in α-position to the NHF warhead. The best compound of the series was inhibitor **44** (Figure 19), which showed hypotensive side effects in a rat study despite its good data in in vitro studies (IC_50_ < 1 nM against *P. aeruginosa* and IC_50_ < 10 nM against *E. coli*) [84].

Two non-hydroxamate compounds selective for LpxC (**45** and **46**, Figure 19) were identified through fragment-based discovery methods. These compounds belong, respectively, to the glycine series and to the imidazole series, fragments that are able to interact with the zinc ion in the catalytic site. Compound **46** showed a promising activity (IC_50_ = 20 nM), and further optimization followed by in vivo studies is ongoing [85].

The imidazole core was also used as starting point by Ushiyama et al. to synthesize a series of inhibitors lacking the hydroxamic acid (HA) moiety. The authors optimized the pharmacokinetic parameters of compound **47** (Figure 19), keeping or improving its antibacterial activity. The study led to the synthesis of compound **48** (Figure 19) and a series of compounds bearing a primary amine in a position adjacent to the imidazole ring. Compound **48** presented inhibitory activity against *K. pneumonie*, including resistant strains, and the onset of cardiovascular diseases decreased with **48** with respect to those compounds bearing a primary amine in a position adjacent to the imidazole [86]. The latter enhanced the antibacterial activity of several antibiotics against Enterobacteriaceae, increasing the bacterial membrane permeability. This synergism could be useful in the treatment of carbapenem-resistant Enterobacteriaceae (CRE) infections [87]. Furthermore, **48** benefited patients with sepsis by acting on the release of lipopolysaccharides (LPS). In any case, further studies will be necessary in order to explain the effect due to the inhibition of LpxC on immune disorders and excessive inflammatory responses [88].

Biological results for the main LpxC inhibitors are summarized in Table 6.

### 2.2. Inhibitors of Pseudolysin and Thermolysin

Inhibitors of pseudolysin also always presented activity on thermolysin due to their high structural homology. For this reason, thermolysin and pseudolysin inhibitors are described in the same section.

#### 2.2.1. Dipicolylamine (DPA) and Tripicolylamine (TPA)-Based Inhibitors

**Figure 20:**
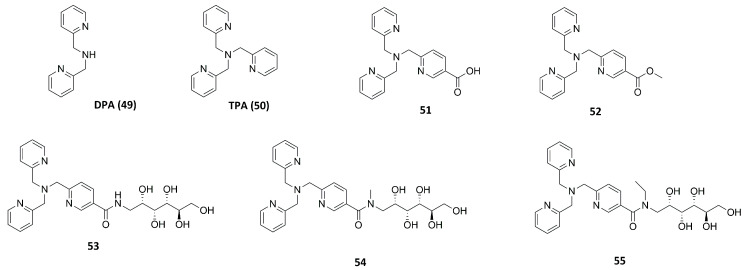
TPA, DPA and their derivatives.

Rahman et al. tested different known compounds as putative inhibitors both on bacterial enzymes (thermolysin (TLN), pseudolysin (PLN) and aureolysin (ALN)) and on human metalloproteinases (matrix metalloproteinase-9 (MMP-9) and matrix metalloproteinase-14 (MMP-14) (Table 7). The tested compounds were DPA (**49**, Figure 20) or TPA (**50**, Figure 20) derivatives already shown to inhibit irreversibly metallo β-lactamases (MBLs) interacting with the Zn^2+^. An initial screening revealed that all of the inhibitors showed a better inhibition of MMP-14 and PLN than of the other enzymes. Furthermore, **50** and **53** (Figure 20) presented strong binding to all enzymes. Compound **51** (Figure 20) and its methyl-ester **52** (Figure 20) presented the same activity against MMP-14 and PLN. Compound **51** was able to coordinate the zinc ion with the carboxylic acid, while **52** coordinated the Zn^2+^ through the nitrogen of the pyridine ring and the methyl-ester group was exposed to solvent. Compounds **53**, **54** and **55** (Figure 20) bearing a hydrophilic methyl-amino glycosyl side chain linked to the **50** core through different amide bonds showed to be weaker than **50**, except for PLN and MMP-14 inhibition. Moreover, Rahman et al. noticed that the substitution of one of the 2-methylpyridine groups of **50** led to a decreasing activity against PLN and MMP-14. Compounds of this series turned out to be reversible inhibitors, in contrast with what is observed for MBLs [37].

#### 2.2.2. Inhibitors with Sulfur-Based ZBGs

##### Thiol Derivatives

**Figure 21:**
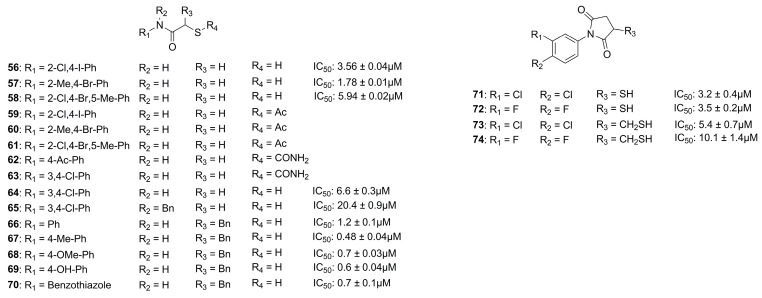
Pseudolysin Inhibitors with sulfur-based ZBGs.

Through a virtual screening study, Zhu et al. identified an aromatic core with a mercaptoacetamide side chain which was able to interact with PLN at non-toxic concentrations. Indeed, they synthesized several compounds and three of them displayed a good activity (**56**, **57** and **58**) (Figure 21). The metalloenzyme selectivity was evaluated in addition to their in vitro activity. Thiol derivative **58** was the only compound inactive against MMP-2 and a Zn^2+^-dependent histone deacetylase (HDAC, encompassing class I and IIb) at a dose range up to 50 µM. The adsorption and metabolism in *Caenorhabditis elegans* were investigated and compounds **56**, **57** and **58** with their respective acetamido prodrugs (**59**, **60** and **61**) were examined. Interestingly, prodrug **61** was the only derivative of this series to be accumulated in the *C. elegans,* metabolized into its activated form (**58**) and then oxidized into the corresponding disulfide analogue. Therefore, prodrug **61** could be considered a good starting point for future studies [89].

**Figure 22:**
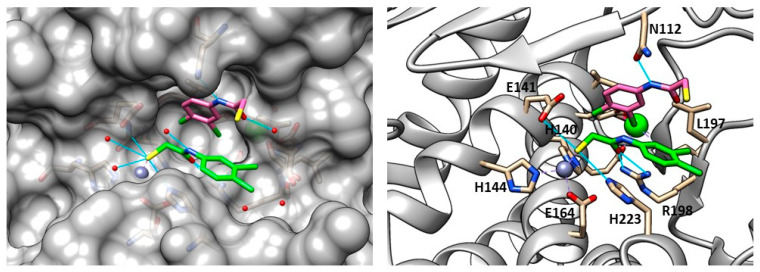
Poses of compound **64** (PDB ID: 6F8B). The green pose chelated the zinc ion, the pink one is linked to a molecular water. Image generated with Chimera, version 1.16.

In 2018, Kany et al. discovered a thiocarbamate derivative (**62**, Figure 21) able to inhibit clostridial collagenase with high selectivity over MMPs. Compound **62** was able to release in vivo its thiol-activated form and to inhibit pseudolysin. Based on the structure of compound **62**, a series of thiocarbamate derivatives has been developed. The best compound was the prodrug **63** (Figure 21), which liberated in vivo the active compound **64** (IC_50_ = 6.6 ± 0.3 µM) (Figure 21). The binding mode of compound **64** is reported in Figure 22. The thiol derivative **64** presented two different binding modes to the pseudolysin catalytic site due to the ambiguous electron density of the binding site. In the pink pose, the zinc ion was not coordinated by the thiol group. Instead, in the green pose the thiol group displaced the water to complete the tetrahedral coordination of the Zn^2+^. Furthermore, the green pose formed a bidentate hydrogen bond with Arg198 in the S1′ subsite and formed another hydrogen bond with the side chain of His223. Otherwise, the amide nitrogen of the pink pose was linked to the Asn112 by a hydrogen bond and its aromatic ring was placed into the S2′ pocket. Neither of the **64** poses led to the closure of the binding site, and for this reason a series of compounds bearing different groups on the amide nitrogen were synthesized, such as compound **65** (IC_50_ = 20.4 ± 0.9 µM) (Figure 21). Unfortunately, none of these derivatives showed improved activity with respect to **64**.

Furthermore, **64** with its prodrug **63** showed an efficacy against IMP-7, a metallo β-lactamase present in clinical isolates of *P. aeruginosa*, with an IC_50_ = 0.86 ± 0.06 µM for **64** and an IC_50_ = 1.16 ± 0.07 µM for **63**, ensuring the future development of novel anti-infective compounds. Finally, the in vitro efficacy towards *Galleria mellonella* infection models was investigated, and the results suggested that the prodrug **63** did not sufficiently release the active thiol **64 [90]**.

**Figure 23:**
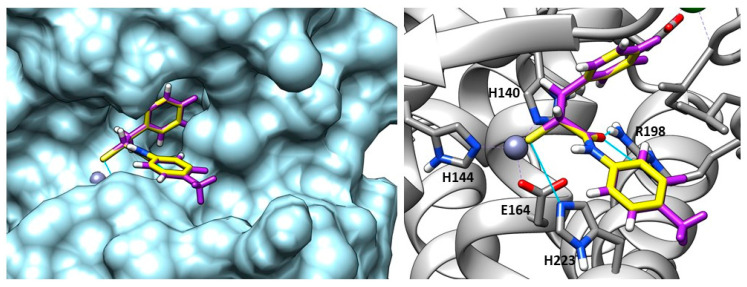
Overlapping between compound **67** (purple) and compound **66** (yellow) into pseudolysin (PDB ID: 7OC7). Image generated with Chimera, version 1.16.

Recently, Kaya et al. synthesized a series of α-benzyl *N-*heteroaryl mercaptoacetamide derivatives as pseudolysin inhibitors. The authors used a structure-based approach on compound **66** (Figure 21), which was previously developed in order to create the new series. Firstly, the binding mode of compound **66** (Figure 23) prompted the idea of increasing the inhibitory activity against pseudolysin by adding a hydrophobic group in the para position of the ring linked to the amide moiety. The introduction of the methyl group led to compound **67** (Figure 21 and Figure 23) with improved hydrophobic interactions with the S1′ pocket. Then, the methyl group of **67** was replaced by a –OMe (compound **68**) and –OH (compound **69**), leading to an improvement of the inhibitory activity.

Furthermore, the same research group replaced the *N*-aryl group with several heterocycles in order to discover new interactions with the surrounding Arg and Asn residues or π–π interactions with the histidine residues. Unfortunately, only benzothiazole derivative **70** (Figure 21) showed a better activity compared to the parent compound **66**, maintaining the selectivity profile over MMPs. The activity of **70** improved thanks to the additional π–π stacking interaction with the histidine residues. In conclusion, the replacement of the *N*-aryl ring with heterocycles did not lead to a significant improvement, so further optimization will be necessary.

Moreover, these inhibitors reduced the pathogenicity of *P. aeruginosa* without affecting the integrity of the lung and skin cells. In addition, **70** (*K*_i_: 0.1 ± 0.01 µM) inhibited *Clostridium histolyticum,* which degrades the collagen fibers, causing several diseases. Further optimization will be necessary to improve the inhibitory efficacy and the physicochemical and pharmacokinetic profiles of thiol derivatives [91].

##### Mercaptosuccinimide Analogues

Konstantinovíc et al. developed a series of compounds bearing a mercaptosuccinimide group as ZBG in order to reduce the flexibility of thiols. These compounds were tested against both pseudolysin and *Clostridium histolyticum* collagenase (ColH). The mercaptosuccinimide analogues were obtained through a structure-based approach starting from compound **64** (Figure 21). The latter showed a nanomolar affinity to ColH in addition to its pseudolysin inhibitory activity. The best compounds, **71** and **72** (Figure 21), showed a better activity than **64**, and compound **72** also displayed a lower toxicity profile than **64**. According to these data, further modification and studies will be relevant in order to obtain good candidates for clinical trials [92]. Imai et al. also proposed two compounds (**73** and **74**, Figure 21) featuring a thiol group not directly linked to the succinimide ring endowed with a lower activity against pseudolysin than their analogues **71** and **72 [51]**.

#### 2.2.3. Hydroxamic Acid-Based Inhibitors

**Figure 24:**
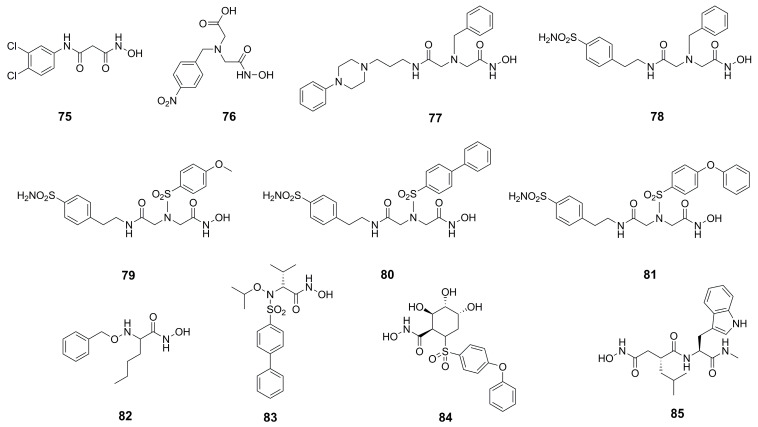
Hydroxamic acid-based inhibitors.

Given the promising results obtained with thiol derivatives in terms of activity and selectivity, Kany et al. maintained the scaffold but replaced the thiol group of their compounds with an hydroxamic acid in order to avoid the oxidation of thiols and their inactivation as disulfide compounds. The introduction of a hydroxamic acid as ZBG led to a decrease of selectivity over MMPs, as expected. However, the group of researchers identified compound **75** (Figure 24), a derivative of **64** (Figure 21), which maintained both the activity against pseudolysin (IC_50_: 17.4 ± 0.8 µM and a *K*_i_: 12.3 ± 0.6 µM) and the selectivity over MMPs. Compound **75** was oriented in the binding site in order to chelate the zinc ion with both the hydroxyl group and the carbonyl group of the hydroxamates moiety. Moreover, the oxygen atom of the hydroxamic acid formed a hydrogen bond with Glu141 and the amide nitrogen interacted with Ala113. The bidentate hydrogen bond between **75** and Arg198 was missing, and this explained the weaker activity of **75** with respect to **64**. Furthermore, the selectivity was retained because **75** did not occupy the S1′ pocket like the classical inhibitors of MMPs. Hence, from these findings it can be deduced that sparing the S1′ pocket represents an element to achieve the selectivity over MMPs [93].

Recently, several MMP inhibitors were tested against pseudolysin and thermolysin by Adekoya et al. In particular, three compounds (**76**, **77** and **78**, Figure 24) displayed a higher affinity for the bacterial enzymes than for human matrix metalloproteinases, as shown in Table 8. These results were confirmed by docking studies where the aforementioned strong affinity was explained by the interaction of the ligands with the S1 and S2′ subpockets. In these regions, there are pivotal differences between the M4 enzymes and the MMPs, demonstrated by the three-dimensional alignment of the enzymes. Indeed, the conserved residue of tyrosine placed in an α-helix of M4 enzymes is located in a loop in the MMPs. Moreover, the Tyr114 (Phe114 for thermolysin) and Trp115 in the bacterial enzymes are replaced by a leucine in the MMPs. The side chain of this leucine as well as the side chain of the previously mentioned conserved tyrosine are pointed outward into the binding site of the MMPs, leading to a narrow access to the S1′ subpocket. This is specific of matrix metalloproteinases compared to M4 enzymes. Hence, these differences in the three-dimensional structure justified the low affinity of compounds **77** and **78** for MMPs. In sharp contrast, the interactions within the S1′ subpocket of compounds **79**–**81** (Figure 24, Table 8) into MMPs contributed to the high affinity to them. In general, these compounds bound better MMPs than bacterial enzymes, and between thermolysin and pseudolysin, the inhibitors presented higher affinity for pseudolysin [18]. These considerations were confirmed later by the same group when they compared hydroxamic acid compounds belonging to different chemical classes.

**Figure 25:**
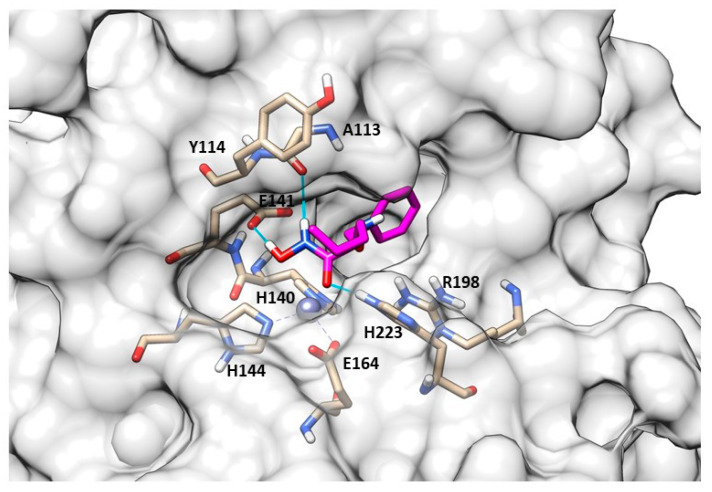
**82** (pink) binding-mode into pseudolysin (PDB ID: 1U4G). Image generated with Chimera, version 1.16.

The best compound of the series was the *N*-benzyloxy-amino acid hydroxamate **82** (Figure 24, Table 8) which showed high activity against pseudolysin and thermolysin compared to MMPs. In particular, **82** displayed better activity against pseudolysin than against thermolysin. In pseudolysin, **82** chelated the Zn^2+^ with the hydroxamic acid group and its benzyl ring was placed within the S2′ pocket while its *n*-butane chain was directed towards the S1′ subsite. The hydrogen of the hydroxyl group participated in a hydrogen bond with Glu141, and the nitrogen atom of the hydroxamate moiety also formed a hydrogen bond with the backbone of Ala113 (Figure 25).

**Figure 26:**
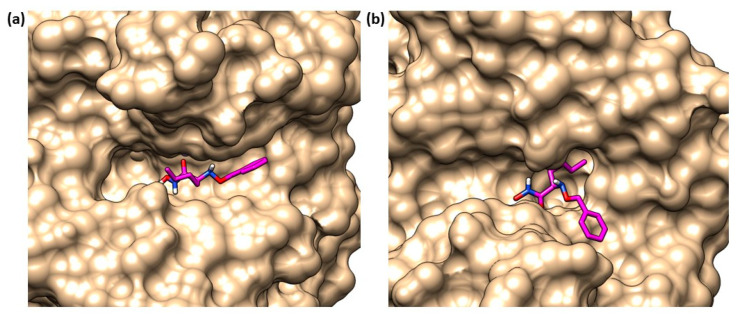
The two possible poses of **82** (pink) in TLN binding site. (**a**) **82** (pink) with its ring in S1 pocket of thermolysin (PDB ID: 1PE5). (**b**) **82** (pink) with its ring in S2′ pocket of thermolysin (PDB ID: 1PE5). Image generated with Chimera, version 1.16.

**Figure 27:**
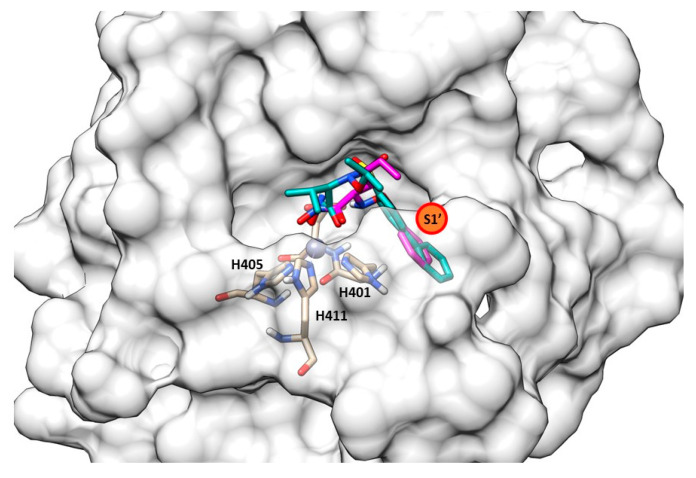
Superposition between **82** (pink) and **83** (light sea-green) into MMP-9 (PDB ID: 1GKC). Image generated with Chimera, version 1.16.

Instead, the binding study did not predict a unique pose for **82** into thermolysin. Compound **82** had two possible poses to inhibit TLN. In one pose, the ring system was in the S2′ pocket (Figure 26b); in the other, the ring system was placed within the S1 subpocket (Figure 26a). However, in both of these poses the zinc ion was coordinated by the hydroxamates through a bidentate chelation. When the ring system was in the S1 subpocket, **82** showed interactions with Phe114 and Asp116 and through its carbonyl group had hydrogen bonds with the Arg203. Additionally, the NH-group interacted with Glu143. Moreover, **82** retained the selectivity towards M4 because it did not fit so deeply into the S1′ pocket or into the S2′ subsite of the MMPs, and the hydroxyl group of the hydroxamic acid also did not form a hydrogen bond with the enzyme (Figure 27). In sharp contrast, sulfonamide derivative **83** showed good IC_50_s towards the endogenous MMPs. These results were validated by docking studies where the hydroxamic acid of **83** chelated the zinc ion and the long ring system was placed deeply in the S1′ pocket (Figure 27).

Thus, it can be deduced that the inhibitory activity against bacterial proteases increases when the ligand has groups that interact strongly within the S2′ subpocket of M4 enzymes [34]. Compounds **84** and **85** (Figure 24, Table 8) were also tested, showing that the size and shape of the inhibitor fragment binding within the S1′ subpocket was a key factor for selectivity. Azasugar sulfonamide **84** was not able to bind the bacterial enzymes due to its big aromatic system, while the known broad-spectrum MMP inhibitor galardin, **85**, inhibited the bacterial enzymes (PLN and TLN) in the nanomolar range (*K*_i_: 20 nM) [94].

#### 2.2.4. Bisphosphonate-, Catechol- and Carboxylate-Based Inhibitors

**Figure 28:**
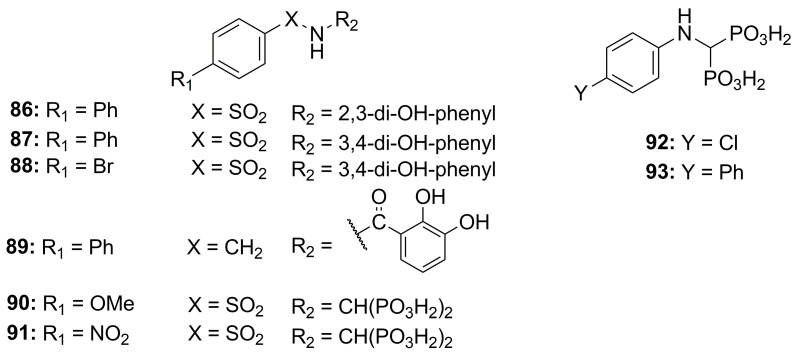
Bisphosphonate-, catechol-based inhibitors.

With the aim of inhibiting bacterial metalloproteases, compounds with different ZBGs were tested. Rahman et al. screened bisphosphonate- and catechol-based inhibitors, previously synthesized to inhibit MMPs, against MMPs (MMP-9 and MMP-14), thermolysin (TLN), pseudolysin (PLN) and aureolysin (ALN). The catechol structures, **86**–**89** (Figure 28, Table 9), did not show strong activity against the bacterial proteases or a good selectivity between bacterial and human metalloproteinases. Instead, the bisphosphonate derivatives (**90**–**93**, Figure 28, Table 9) displayed a high affinity for pseudolysin and thermolysin compared to the catechol analogues. This inhibitory activity was achieved by the interaction between one of the bisphosphonate groups and the arginine residue (Arg198 for PLN, Arg203 for TLN) located at the entrance of the S1 subpocket [95].

**Figure 29:**
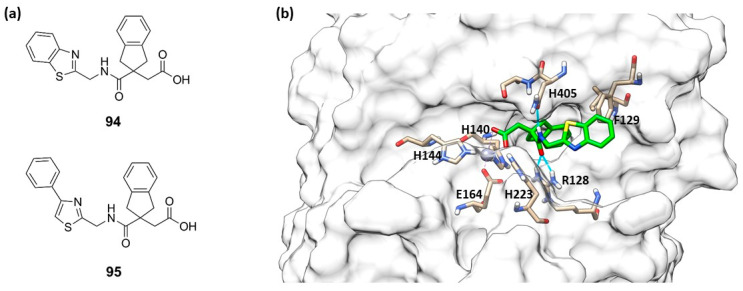
(**a**) Structures of **94** and **95**. (**b**) Compound **94** (green) within the catalytic site of pseudolysin (PDB ID: 7AJR). Image generated with Chimera, version 1.16.

Leiris et al., through a virtual screening and computer-assisted drug design, identified two carboxylic acids (**94** and **95**, Figure 29a) that were able to inhibit the *P. aeruginosa* elastase. These indane carboxylic acids, **94** and **95**, inhibited pseudolysin with a *K*_i_ of 0.16 µM and 0.12 µM, respectively. Compound **94** formed a strong interaction both with Arg198 and Asn112 through a bidentate hydrogen bond. Additionally, the benzothiazole was placed into a cleft closed to the S2′ pocket by π–π interactions with Phe129. Furthermore, carboxylic acid **94** extended its interaction to the base of the S2′ pocket mediated by a binding with a water molecule (Figure 29b) [96]. Further investigations will be necessary for delivering an innovative antibacterial drug.

Biological results for the main PLN and TLN inhibitors are summarized in Table 10.

## 3. Conclusions

Nowadays, infections due to Gram-negative bacteria are emphasized by the increasing multidrug-resistance phenomenon. Inhibition of bacterial metalloenzymes LpxC, pseudolysin and thermolysin may represent an alternative strategy to combat the development of infections caused by Gram-negative bacteria, avoiding the resistance mechanism. The inhibition of LpxC interferes with the bacterial outer membrane permeability, enhancing drug absorption, whereas the inhibition of pseudolysin and thermolysin affects the bacterial nutrition and sporulation.

In this review, we presented all the synthetic metalloenzymes inhibitors against LpxC, PLN and TLN reported in literature from 2015 to the present. These inhibitors have been classified based on their target protein and according to their chemical structure.

LpxC inhibitors have been divided into hydroxamic acid-based (HA) inhibitors and non-hydroxamate inhibitors. Among HA, compound **4** was the hit compound which inspired another series of inhibitors.

The hydroxamic acid **14** with a di-acetylene tail (IC_50_ = 0.68 nM) was the first inhibitor to reach Phase I of clinical trials. Moreover, among the series developed by Novartis, compound **20** (IC_50_ = 1.5 nM), bearing a propargyl group in its tail, displayed good results both in vitro and in vivo, and further studies are ongoing.

Several compounds belonging to the *C*-furanose class have also shown good activity. The best compound of this series was **42** (IC_50_ = 8.5 ± 1.3 µM), and its higher activity was due to the interaction between the triazole ring and Lys239 and Phe192 residues. However, more studies will be required to analyze the safety of these promising compounds. Furthermore, among compounds without hydroxamic acid as ZBG, the best compound **48** (IC_50_ = 0.101 µM) showed several benefits in patients with cardiovascular diseases or in sepsis condition. Biological results for the main LpxC inhibitors have finally been summarized in Table 6.

Inhibitors against PLN and TLN have been classified based on their ZBG. Among the thiol derivatives, the pro-drug **61** was shown to be a promising candidate due to its capacity to release compound **58** (IC_50_ = 5.94 ± 0.02 µM) active against *C. elegans*. Moreover, the α-benzyl *N*-heteroaryl mercaptoacetamide compound **67** (IC_50_ = 0.48 ± 0.04 µM) showed a good activity against PLN due to the interaction of the ring linked to the amide moiety within the S1′ subpocket.

The best results were reached by those inhibitors bearing the HA as ZBG. Several MMP inhibitors, previously synthesized, were tested against M4 enzymes. Among these, compound **82** showed higher affinity against PLN (IC_50_ = 1.9 µM) and TLN (IC_50_ = 9.5 µM) than against MMPs. This selectivity was achieved because **82** did not fit so deeply into the S1′ pocket or into S2′ subsite of the MMPs. Biological results for the main PLN and TLN inhibitors have been summarized in Table 10.

Based on the results shown, LpxC inhibitors hold promising potential as new antibacterial agents. Future research should aim to optimize the properties of LpxC inhibitors to ensure good bioavailability and pharmacokinetics. Otherwise, research involving PLN and TLN inhibitors is less developed, and further studies are required to improve both enzymatic efficacy and pharmacokinetic properties of these agents.

## Figures and Tables

**Table 1:** Bacteria and their typical diseases. Adapted from [10].

Name of Bacterium	Drug(s) Resistant to	Typical Diseases
*Escherichia coli*	Cephalosporins and fluoroquinolones	Urinary tract infections and blood stream infections
*Klebsiella pneumonia*	Cephalosporins and carbapenems	Pneumonia, blood stream, and urinary tract infections
*Staphylococcus aureus*	Methicillin	Wound and blood stream infections
*Streptococcus pneumoniae*	Penicillin	Pneumonia, meningitis, and otitis
Nontyphoidal *salmonella*	Fluoroquinolones	Foodborne diarrhea, blood stream infections
*Pseudomonas aeruginosa*	Carbapenems	Chronic ulcers, lung infections
*Shigella species*	Fluoroquinolones	Diarrhea (bacillary dysentery)
*Neisseria gonorrhoeae*	Cephalosporins	Gonorrhea
*Mycobacterium tuberculosis*	Rifampicin, isoniazid, and fluoroquinolone	Tuberculosis

**Table 2:** MIC values of Surivet LpxC inhibitors.

Compound	MIC (µg/mL) *E. coli*	MIC (µg/mL) *K. pneumoniae*	MIC (µg/mL) *P. aeruginosa*
**8**	0.25	1	0.5
**9**	0.12	0.25	0.5
**10**	0.5	0.5	0.5
**11**	0.5	1	1

**Table 3:** Inhibitory activity of compounds **4**, **12** and **13**.

Compound	*E. coli* IC_50_ (nM)	MIC (µg/mL) *E. coli*	MIC (µg/mL) *K. pneumoniae*	MIC (µg/mL) *P. aeruginosa*
**4**	3	0.125	0.25	0.5
**12**	1200	2	64	1
**13**	12	0.063	0.5	0.5

**Table 4:** Inhibition activity of compounds **28**–**32**.

Compound	Configuration	Zone of Inhibition [mm]		Enzyme Assay	
		*E. coli BLB21 (D3)*	*E. coli D22*	IC_50_ [µM]	K_i_ [µM]
**28**	(2S, 3R, 4S, 5S)	<6	13.7 ± 1.8	>200	>27.6
**29**	(S, S)	11.5 ± 1.5	24.2 ± 1.5	2.6 ± 0.3	0.36 ± 0.04
**30**	(S, S)	13.7 ± 0.6	21.3 ± 2.5	1.7 ± 0.4	0.23 ± 0.05
**31**	(2S, 3S, 4R, 5S)	22.3 ± 1.4	28.3 ± 1.4	3.2 ± 1.0	0.4 ± 0.1
**32**	(2S, 3R, 5R)	18.0 ± 1.0	25.3 ± 1.5	23.7 ± 17.6	3.5 ± 2.4

**Table 5:** Inhibition activity of acyclic series E.

Compound	Configuration	Zone of Inhibition [mm]		Enzyme Assay	
		*E. coli BLB21 (DE3)*	*E. coli D22*	IC_50_ [µM]	K_i_ [µM]
**29**	(S, S)	9.0 ± 0.5	20.8 ± 0.6	2.6 ± 0.3	0.36 ± 0.04
**33**	(S)	9.5 ± 0.4	20.5 ± 0.2	0.48 ± 0.23	0.066 ± 0.032
**34**	(S)	13.4 ± 0.5	21.2 ± 0.6	0.69 ± 0.30	0.095 ± 0.042
**35**	(R)	9.1 ± 0.4	13.0 ± 1.7	31.6 ± 6.0	4.4 ± 0.8
**36**	(R)	8.7 ± 0.7	12.3 ± 1.6	198 ± 12	27.3 ± 1.7
**37**	(S)	11.7 ± 0.6	20.7 ± 1.7	1.96 ± 0.36	0.27 ± 0.05
**38**	(S)	15.7 ± 0.6	25.8 ± 1.9	2.82 ± 0.5	0.39 ± 0.07
**39**	(R)	10.3 ± 2.5	17.0 ± 1.0	1.87 ± 0.85	0.26 ± 0.12
**40**	(R)	12.3 ± 0.6	22.0 ± 1.3	1.66 ± 0.31	0.23 ± 0.04

**Table 6:** Inhibitory activity (IC_50_ nM) of the main LpxC inhibitors.

Compound	LpxC	Bacterial Strain	Ref.
**4**	3	*E. coli*	[68]
**14**	0.68	*P. aeruginosa*	[69]
**15**	0.71	*P. aeruginosa*	[69]
**20**	1.5	*P. aeruginosa*	[60]
**22**	6	*E. coli*	[73]
**42**	8500	*E. coli*	[79]
**46**	20	*P. aeruginosa*	[85]

**Table 7:** *K*_i_ ± s.d. (µM) values of TPA, DPA and their derivatives for MMP-14, MMP-9, ALN, PLN and TLN.

Compound	MMP-14	MMP-9	ALN	PLN	TLN
**49**	n.d.	n.d.	n.d.	4 ± 1	n.d.
**50**	1.2 ± 0.1	15 ± 4	16 ± 1	5 ± 2	5.5 ± 0.9
**51**	3.8 ± 0.3	21 ± 5	25 ± 2	4 ± 1	n.d. *
**52**	1.5 ± 0.1	n.d.	n.d.	12 ± 4	n.d.
**53**	3.5 ± 0.3	22 ± 6	20 ± 2	1.1 ± 0.3	12 ± 2
**54**	8.6 ± 0.7	n.d.	n.d.	5 ± 2	n.d.
**55**	4.5 ± 0.4	28 ± 7	n.d.	5 ± 2	9 ± 2

* n.d., not determined.

**Table 8:** IC_50_ (µM) values of hydroxamic acid derivatives **76**–**83**.

Compound	Thermolysin	Pseudolysin		MMP-1	MMP-2	MMP-9	MMP-14	ADAM-17
	BLS	BLS	AGLA					
**76**	755 ± 17	n.d. *	20 ± 2	n.d.	178 ± 6	>300	313 ± 25	-
**77**	8 ± 1	2.8 ± 0.2	6.7 ± 0.5	n.d.	>300	>300	>300	-
**78**	12 ± 1	n.d.	0.4 ± 0.3	n.d.	>100	>300	>100	-
**79**	164 ± 22	33 ± 2	15 ± 2	0.53 ± 0.02	0.025 ± 0.002	0.054 ± 0.003	0.090 ± 0.004	-
**80**	67 ± 4	n.d.	31 ± 13	0.54 ± 0.02	0.016 ± 0.001	0.066 ± 0.006	0.26 ± 0.02	-
**81**	160 ± 31	276 ± 18	16 ± 2	0.14 ± 0.01	0.0016 ± 0.0003	0.00051 ± 0.00004	0.0021 ± 0.0002	-
**82**	9.5	1.9	1.1	-	34	81	-	100
**83**	800	798	57	-	0.0008	0.0067	-	14

* n.d and -, not determined. BLS: the bradykinin-like substrate. AGLA: the substrate Abz-Ala-Gly-Leu-Ala-p-nitrobenzylamina.

**Table 9:** *K*_i_ values of catechol and bisphosphonate compounds.

	*K*_i_ ± sd (µM)				
Compound	TLN	PLN	ALN	MMP-14	MMP-9(T)
**86**	n.d. *	38 ± 8	n.d.	19 ± 0.8	51 ± 17
**87**	13 ± 2	9 ± 3	49 ± 5	6.6 ± 0.6	13 ± 2
**88**	14 ± 5	16 ± 3	n.d.	8.3 ± 0.6	12.6 ± 0.6
**89**	n.d.	n.d.	n.d.	12 ± 1	13 ± 1
**90**	n.d.	58 ± 4	n.d.	n.d.	n.d.
**91**	16.2 ± 0.4	22 ± 3	n.d.	n.d.	n.d.
**92**	n.d.	37 ± 6	n.d.	17 ± 1	n.d.
**93**	7 ± 1	12 ± 4	n.d.	7.2 ± 0.6	6.6 ± 0.4

* n.d.: not determined.

**Table 10:** Inhibitory activity (IC_50_ µM) of the main zinc metalloproteinase inhibitors against PLN, TLN, MMP-2 and MMP-9.

Compound	PLN	TLN	MMP-2	MMP-9	Ref.
**57**	1.78 ± 0.01	- ^a^	-	-	[89]
**66**	1.2 ± 0.1	-	-	-	[91]
**67**	0.48 ± 0.04	-	-	-	[91]
**68**	0.7 ± 0.03	-	-	-	[91]
**69**	0.6 ± 0.04	-	-	-	[91]
**78**	0.4 ± 0.3	12 ± 1	>100	>300	[18]
**82**	1.5 ^b^	9.5	34	81	[34]

^a^ “-”: not tested or unknown from the corresponding original reference. ^b^: This value is an average between two IC_50_ values calculated on two different substrates (BLS and AGLA) [34].

## Data Availability

No new data were created or analyzed in this study. Data sharing is not applicable to this article.
